# Bacterial Communication: The Possible Role of Quorum Sensing, Quantum Mechanics, and Quantum Tunneling

**DOI:** 10.3390/metabo16080542

**Published:** 2026-07-31

**Authors:** Leon M. T. Dicks, Carolina Pohl, Alfred Botha

**Affiliations:** 1 Department of Microbiology, Stellenbosch University, Stellenbosch 7600, South Africa; abo@sun.ac.za; 2 Department of Microbiology and Biochemistry, University of the Free State, Bloemfontein 9300, South Africa; pohlch@ufs.ac.za

**Keywords:** bacterial communication, quorum sensing, quantum mechanics, quantum tunneling

## Abstract

**Highlights:**

- Quantum mechanics, and not only quorum sensing, may play a role in bacterial communications.
- Quantum tunneling might contribute to the crossing of ions across cell membranes, in addition to ions passing through protein channels.
- The long-range signaling ability of quanta could, hypothetically, allow bacterial cells to maintain contact over long distances, modify their metabolic activities, and activate DNA repair systems.
- The wave-like behavior of subatomic particles and molecules causes nanovibrations and generates electromagnetic fields, as shown for photons. Experimental evidence that bacteria use quantum mechanics to communicate is lacking, although theoretically possible and requires further research.

**Abstract:**

Bacteria in the human intestinal tract express more than 46 million genes, suggesting that interbacterial communication and communication with human cells are well-controlled and synchronized. Many papers have been published on quorum sensing (QS) and other forms of bacterial communication, e.g., nanotubes, nanovibrations, and electromagnetism. Autoinducers (AIs) such as AI-1 N-acyl homoserine lactones (AHLs), AI-2 boron-containing furanosyl borate diesters, AI-3 pyrazinone derivatives, a combination of AI-3/Epi (epinephrine)/NE (norepinephrine), auto-inducer peptides (AIPs), and SdiA (suppressor of division inhibition), along with their receptors, have been well-studied. However, little is known about the roles of quantum mechanics, quantum tunneling, and quantum entanglement in bacterial communication. Most proposals are hypothetical and remain conceptual frameworks supported by indirect evidence rather than validated experiments. The wave-like behavior of ions (quantum tunneling) may facilitate crossing potential energy barriers, such as cell membranes, in concert with protein channels. If this is indeed the case, ions in a quantum-tunneling state would, hypothetically, be able to pass through any part of the cell membrane and cell wall. This would, in theory, enhance biochemical reactions, interbacterial communication, and interactions with human cells. The long-range signaling ability of quanta could allow bacterial cells to maintain contact over long distances, modify their metabolic activities, and activate DNA repair systems. The wave-like behavior of subatomic particles and molecules may cause nanovibrations and generate electromagnetic fields. We argue that electrical signals generated within a biofilm by quanta may attract distant cells and enable cross-species communication. We propose a hypothetical “two-pillar” bacterial communication system, i.e., QS and quantum mechanics/tunneling/entanglement (QMTE), and discuss the advantages of combining both.

## 1. Introduction

Humans harbor approximately 3000 species of gut microbiota, of which the majority (close to 90%) are bacteria [1,2]. More than 46 million genes are encoded by approximately 5 × 10^13^ bacteria, which contribute to over 99% of the genetic material in our bodies [3,4]. With so many genes involved, communication among the gut microbiota must be extensive, highly organized, and synchronized to exert such a profound influence on our physiology, intellect, emotions, cognition, consciousness, and decision-making processes.

**Figure 1:**
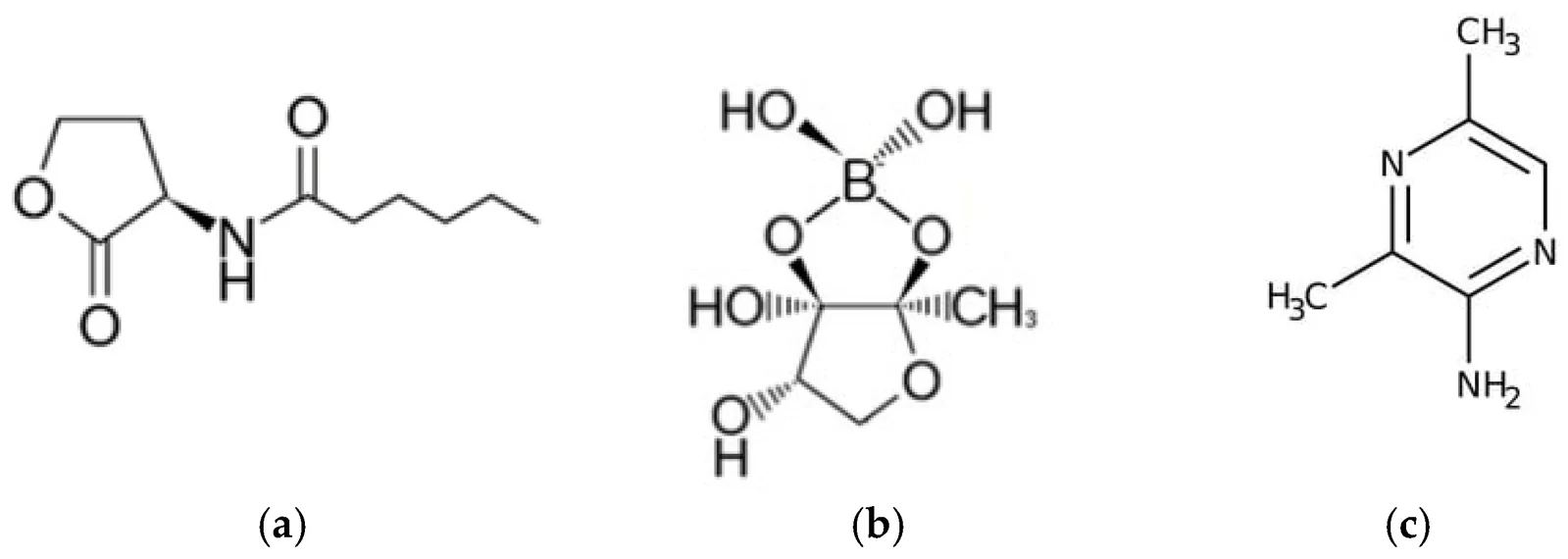
Structure of autoinducers: (**a**) Al-1 (unsubstituted homoserine lactone, C6 HSL); (**b**) Al-2 (furanosyl borate diester); (**c**) Al-3 (a pyrazinone, e.g., phevalin).

Most scientific papers describe intra-bacterial communication and communication between bacteria and humans based on quorum sensing (QS). The three main QS autoinducers are AI-1 N-acyl homoserine lactones (AHLs), which are lipid molecules with a homoserine lactone (HSL) moiety linked to a 4- to 18-carbon acyl side chain; AI-2 furanosyl borate diesters; and AI-3 pyrazinone derivatives, e.g., 3,5-dimethylpyrazin-2-amine (Figure 1) [1,2]. QS serves as a chemical “language” that bacteria use to communicate with one another and with higher life forms. Other forms of bacterial communication include direct (physical) contact via nanotubes [5,6] and different waves, e.g., nanovibrations, acoustic signals, and electromagnetic fields. This will not be covered in this review. Abiotic (biophysical) forces are usually discussed separately from bacterial communication, which focuses on biochemical pathways and biochemical domains. For further reading on biophysical communication related to mechanical and electric stimuli, magnetic fields, radiation, and theromosensing, the reader is referred to the review by de la Viuda et al. [7].

Classical QS alone may not fully explain bacterial communication because signaling molecules tend to diffuse in complex fluidic environments [8]. Moreover, bacteria detect and decode signaling molecules from entirely different species [1]. In mixed cell populations, competition for nutrients may alter microenvironments before the minimal QS threshold is reached [9]. In addition, host organisms can mimic, degrade, or block signal molecules and disrupt cell coordination [1,10].

In this review, we address the question that most scientific papers on bacterial communication do not cover: Can bacteria communicate with each other and with human cells via quantum mechanical mechanisms, including quantum entanglement? The first part of the paper summarizes QS, the primary communication system used by bacteria. The second part summarizes quantum communication and the roles of quantum tunneling and entanglement in cellular processes, based on recent scientific findings. We discuss the potential advantages of quantum behavior in bacterial communication.

## 2. Quorum Sensing (QS)

**Figure 2:**
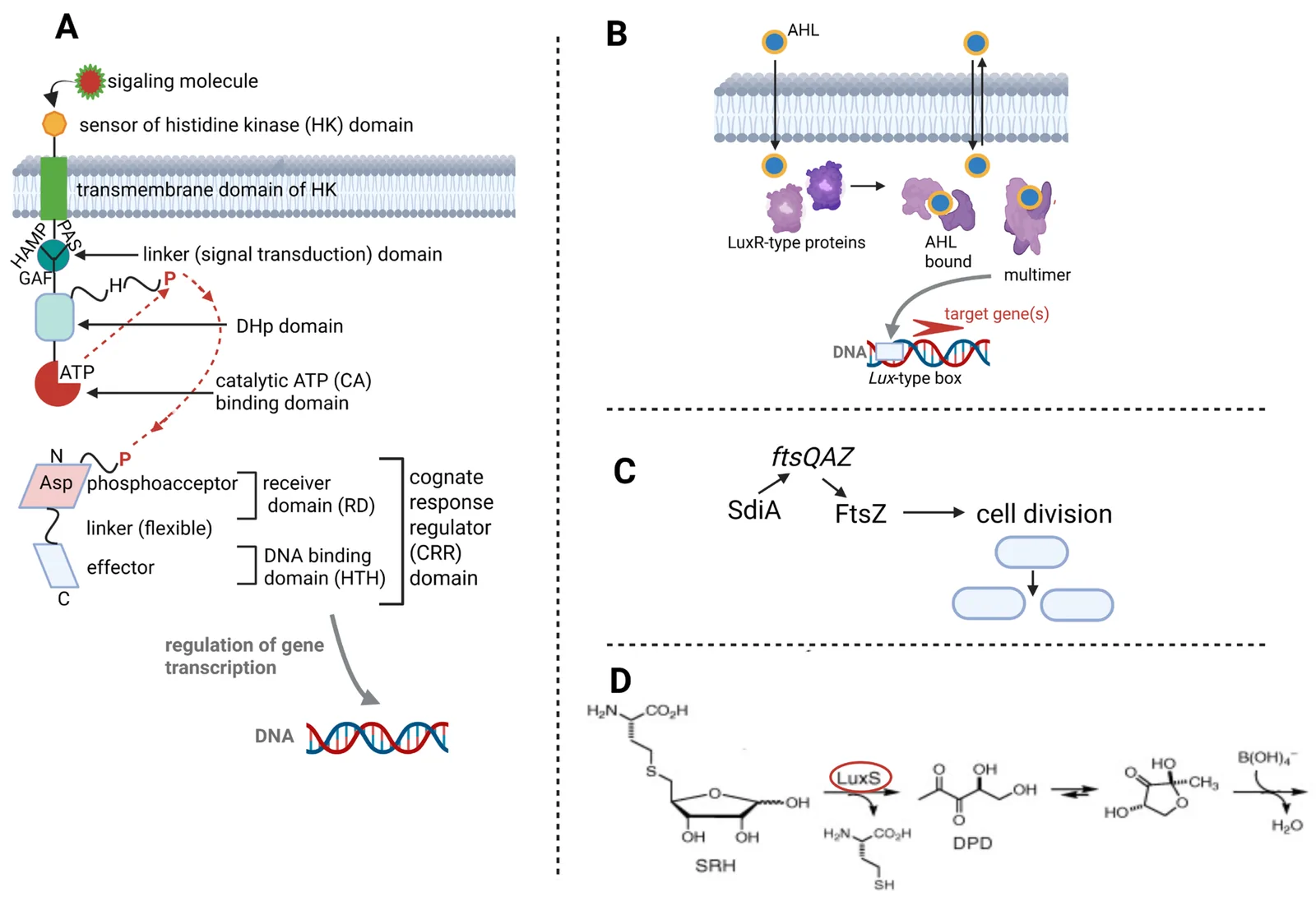
(**A**) Autoinducers (AIs) of Gram-negative bacteria, here shown as a signaling molecule (red dot in the left pane), link to a transmembrane two-component system (TCS) composed of a histidine kinase (HK) domain, a signal transduction domain (linker), a dimerized histidine phosphotransferase (DHp) domain, and a catalytic (CA) domain. Energized phosphate groups are transferred from the DHp and CA domains to the receiver domain (RD) and activate the DNA-binding domain (HTH). (**B**) N-acyl homoserine lactones (AHLs) of Gram-negative bacteria pass through the cell membrane, bind to soluble receptor proteins (LuxR, luciferase regulators), and form AHL-protein complexes that bind to promoters that turn genes on or off. (**C**) SdiA (suppressor of division inhibition) is a QS receptor that activates transcription of the *ftsQAZ* gene cluster, thereby increasing the production of the cell-division protein FtsZ, allowing cells to divide. (**D**) LuxS (S-ribosylhomocysteine lyase) cleaves the thioether bond of S-ribosylhomocysteine (SRH) to produce 4,5-dihydroxy-2,3-pentanedione (DPD), which, due to its instability, immediately cyclizes and rearranges into various AI-2 derivatives. The figure was prepared using BioRender.com (accessed 2 June 2026).

The autoinducers (AIs) in Gram-negative bacteria target transcription factors or transmembrane two-component systems (TCSs) composed of histidine sensor kinases (Figure 2A) [11].

**Figure 3:**
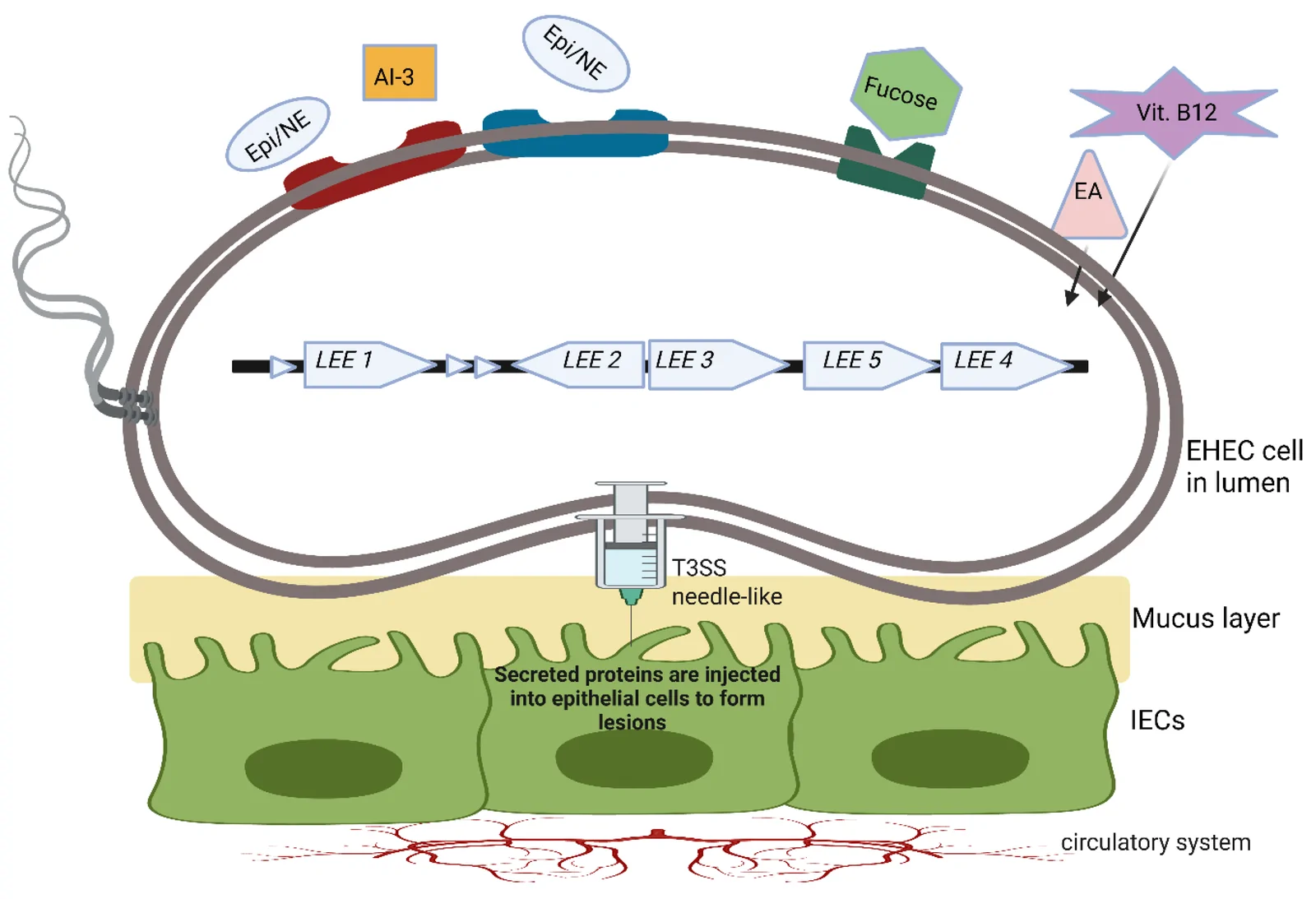
Enterohemorrhagic *E. coli* (EHEC) uses the AI-3/Epi/NE (autoinducer 3/epinephrine/norepinephrine) pathway to upregulate the locus of enterocyte effacement (LEE) pathogenicity island, promoting the formation of lesions in intestinal epithelial cells (IECs). AI-3 binding to Epi and NE increases virulence. During IEC infection, Epi and NE leak into the gut lumen. Bacterial cells sensing AI-3, Epi, NE, fucose, ethanolamine (EA), and vitamin B12 activate transmembrane histidine kinase (HK) receptors (indicated here as rectangles). A more detailed version of this diagram can be found in Dicks [1]. The figure was prepared using BioRender.com (accessed 2 June 2026).

Gram-negative bacteria may produce several forms of AHLs [12], each detected by specific LuxR (luciferase regulator) receptors [13,14]. AHLs pass through the cell membrane and bind directly to soluble receptor proteins (Figure 2B). The SdiA (suppressor of division inhibition) QS receptor, found in *Escherichia coli* and *Salmonella* spp., activates transcription of the *ftsQAZ* gene cluster, thereby increasing production of the cell-division protein FtsZ (Figure 2C). LuxS (S-ribosylhomocysteine lyase) cleaves the thioether bond of S-ribosylhomocysteine (SRH) to produce 4,5-dihydroxy-2,3-pentanedione (DPD), which, due to its instability, immediately cyclizes and rearranges into various AI-2 derivatives (Figure 2D). AI-3, combined with Epi and NE, forms the AI-3/Epi/NE pathway (Figure 3), which coordinates gene expression, motility, and virulence [15]. Enterohemorrhagic *E. coli* (EHEC) uses the AI-3/Epi/NE pathway to upregulate the locus of enterocyte effacement (LEE) pathogenicity island, promoting the formation of lesions in intestinal epithelial cells (IECs) [16]. For further information, the reader is referred to the review by Dicks [1].

Gram-positive bacteria communicate via small linear or cyclized oligopeptides (QS peptides (QSPs), also referred to as autoinducing peptides (AIPs)). The pentapeptide CSF (competence sporulation factor) and the heptapeptide SDLPFEH (PapRIV) are well-studied [17,18]. These peptides interact with either membrane-localized receptors or cytoplasmic sensors such as proteins Rap, NprR, PlcR, and PrgX24. In a TCS, the AIP binds to a sensor on the surface of the receiving cell that is linked to the N-terminus of a histidine kinase (HK) [19]. For more information on QSPs, the reader is referred to the Quor-umpeps^®^ database [20].

## 3. Quantum Mechanics and Quantum Tunneling

**Figure 4:**
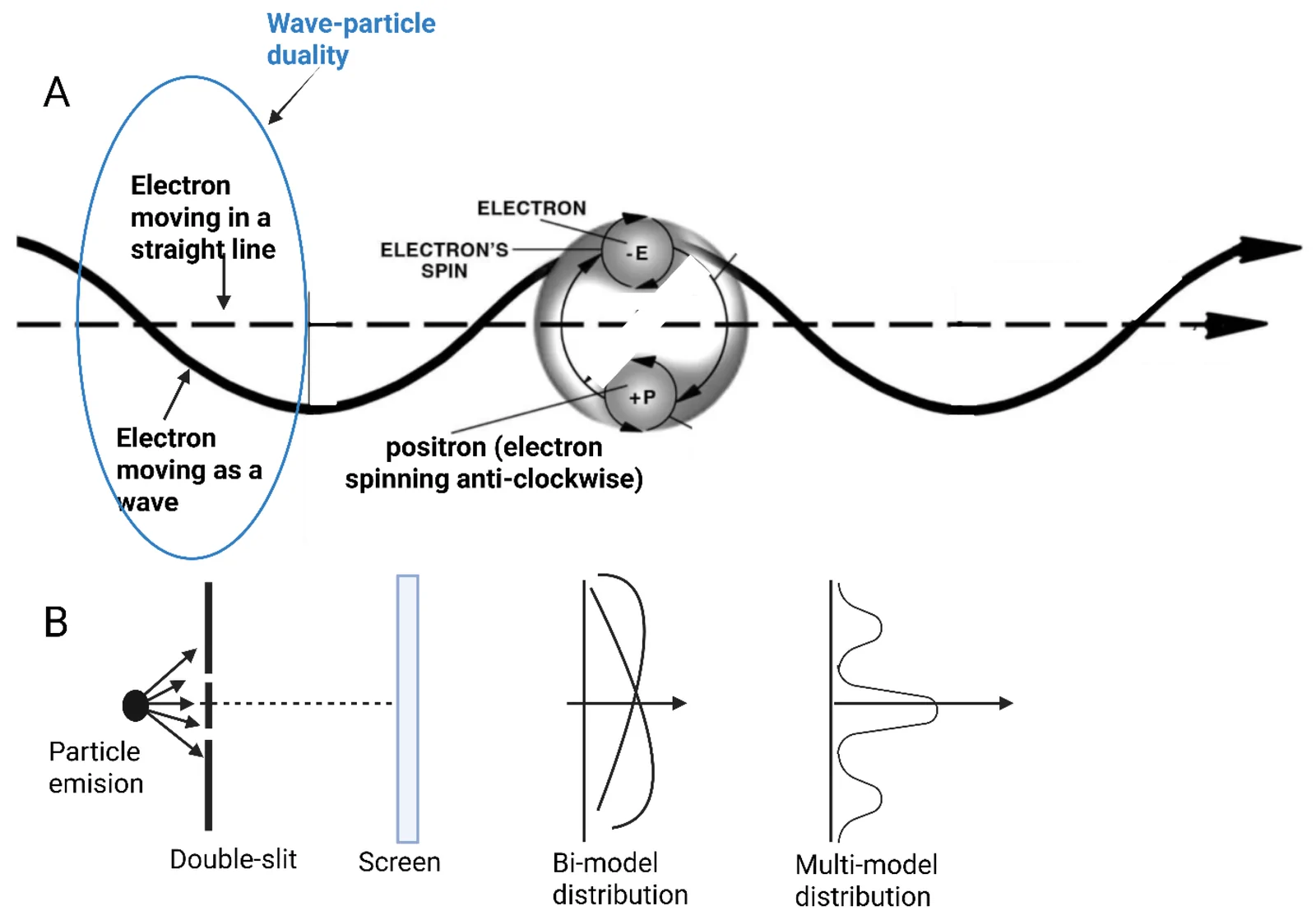
Modulated two-dimensional behavior of electrons, protons, neutrons, atoms, and photons, showing their movement in straight lines (linear and localized) or as waves (spread-out) (**diagram A**). For the sake of simplicity, the movement of electrons is described. Based on this model, wave–particle duality allows particles to simultaneously exist as discrete entities and participate in two separate chemical reactions. Wave–particle duality was first demonstrated with photons moving through a double slit (**diagram B**). When single photons, one at a time, pass through two slits (representing a barrier or cell membrane as we propose), they distribute to form either a bimodal pattern (observed as a single line) or a multimodal interference pattern. The figure was prepared using BioRender.com (accessed 3 June 2026).

Quantum mechanics describes how subatomic particles such as electrons, ions, protons, neutrons, and photons, as well as atoms, behave. A quantum is the smallest possible, indivisible unit of any physical property, such as energy or matter. The discrete packets in which electromagnetic energy is absorbed or emitted are referred to as quanta. Subatomic and atomic particles exhibit two-dimensional behavior: they move in straight lines (linear and localized) or as waves (spread out), as modulated in Figure 4A [21]. Wave–particle duality (quantum tunneling) allows particles to simultaneously exist as discrete entities and participate in two separate chemical reactions. Quantum tunneling was first demonstrated in 1971 with photons passing through a double slit, in what is referred to as “Parker’s single-photon double-slit experiment” [22]. When single photons, one at a time, pass through two slits (representing a barrier), they distribute in such a way that they form either a bimodal pattern (visible as a single line) or a multimodal interference (distribution) pattern (Figure 4B). The behavior of the waves is, as expected, influenced by the distance between the slits [23]. If we apply the concept of the double-slit experiment to ion channels in bacterial cell membranes, subatomic and atomic particles passing through open channels may form bimodal or multimodal interference patterns and exhibit quantum interference, as illustrated in Figure 4B. Energy, charge, or information transfer are important phenomena in physical and biological systems and occur at scales ranging from atoms to large macromolecular structures. Quantum mechanics may influence the efficiency at which energy and charged ions are transported through the cell membrane. Granted, at a physiological temperature and in a wet environment, such as the cytoplasm of a bacterial cell, where particles collide with water molecules, lipids, and other ions, they behave in a state of decoherence, even if only for a few picoseconds. Thus, ions passing through a membrane channel are expected to be in a scattered (decohered) state and to lose some of their energy.

Quantum biology faces major limitations in living systems due to thermal noise, rapid decoherence, and the extreme challenge of proving long-range quantum communication in bacteria. This random thermal motion causes rapid decoherence, destroying delicate quantum superpositions and entanglements in picoseconds. Dynamic, highly active, and rapidly dividing cells, such as bacteria, communicate via QS. Any non-classical mechanisms of communication, based on quantum mechanics, must be distinguished from standard chemical and electrical signals. However, this does not mean quantum physics can be ruled out as a method of bacterial communication.

The selectivity of ion channels in cell membranes has been studied for more than 50 years, but several questions remain unanswered. In a modulated study, Salari et al. [24] suggested that quantum interference may explain ion channel selectivity, since interference only occurs between similar ions through the same-sized ion channels in a cell membrane. They have shown that, on average, the quantum state of an ion (such as K^+^) in a cell membrane channel survives for approximately 100 picoseconds and, at most, 53 picoseconds outside the channel [23,24] (thus, when secreted). The maintenance of an ion’s quantum state after passing through the membrane channel suggests that ions (and possibly other subatomic and atomic particles, or even molecules and small peptides) move across cell membranes due to quantum interference. From this, we deduce that quantum decoherence, as expected in a biological environment, does not destroy an ion’s velocity. It is conceivable that the level of quantum energy retained by the secreted ion will be determined by its original energy before passing through the channel, the selectivity of the proteins forming the channel (which depends on the amino acid charges), and interference from other particles that also flow through the channel. In fact, Salari et al. [24] have shown that quantum interference increases the transport of K^+^ to approximately 10^8^ ions per second. To resist quantum interference for approximately 100 picoseconds, the channel must be at least 5.9 nm in diameter [24]. The movement of K^+^ through potassium channels is much faster than the calculated 10^2^ to 10^4^ ions per second achieved by a sodium–potassium pump (Na^+^/K^+^-ATPase) [25]. The movement of K^+^ through potassium channels drives long-range electrical signaling, metabolic coordination (nutrient sharing), and intercellular communication in bacterial communities, especially in biofilms [26]. This may explain how bacteria adapt to rapidly changing environments but needs to be confirmed with biological studies.

**Figure 5:**
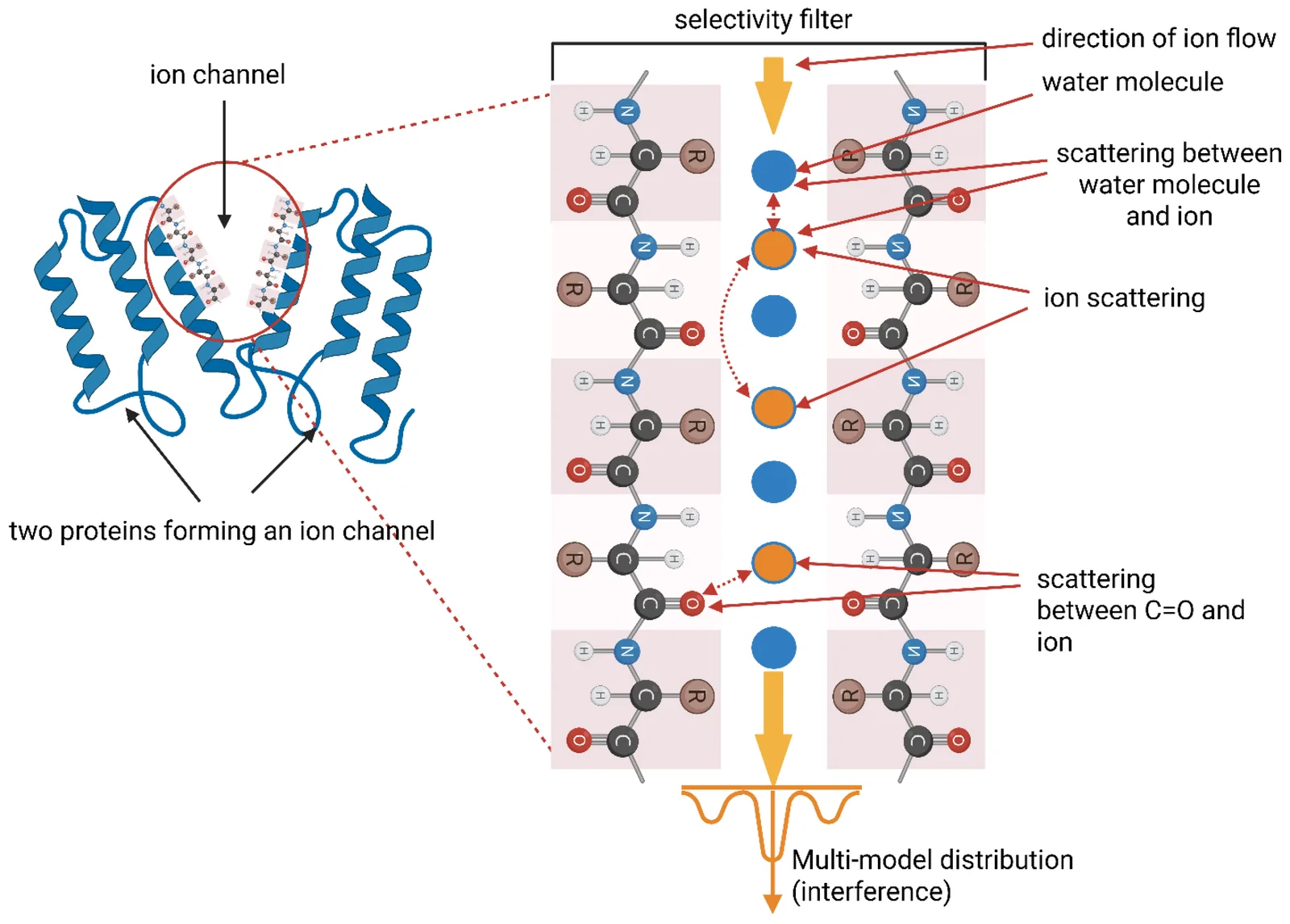
A schematic representation showing the hypothetical movement of K^+^ through a potassium channel formed by two proteins. The amino acid sequence depicted is for explanatory reasons and not specific to a potassium ion channel. In a K^+^ channel of approximately 1.2 nm long, the protein “filter” is composed of four phosphate (P) loops. The carbonyl groups (C=O’s) trap and displace the ions. The quantum states of ions in the channel are determined by the charges on the amino acids and by collisions with other particles in the channel (e.g., water molecules). Each particle (in this example, K^+^) behaves as a delocalized wave, passing simultaneously through the channel. These waves superimpose, creating a dense, multi-peaked interference pattern. The figure was prepared using BioRender.com (accessed 3 June 2026).

The composition of a protein channel, showing the hypothetical movement of ions through the channel, is shown in Figure 5. The proteins forming the membrane channel enable highly selective ion conduction. Each ion channel is specialized for specific ions, depending on the arrangement of amino acids. Potassium channels permit only K^+^ and prevent the passage of Na^+^. This “selectivity filter” is characteristic of each cell membrane channel [24].

## 4. How Do Quantum Mechanical Laws Apply to Bacterial Cells?

**Figure 6:**
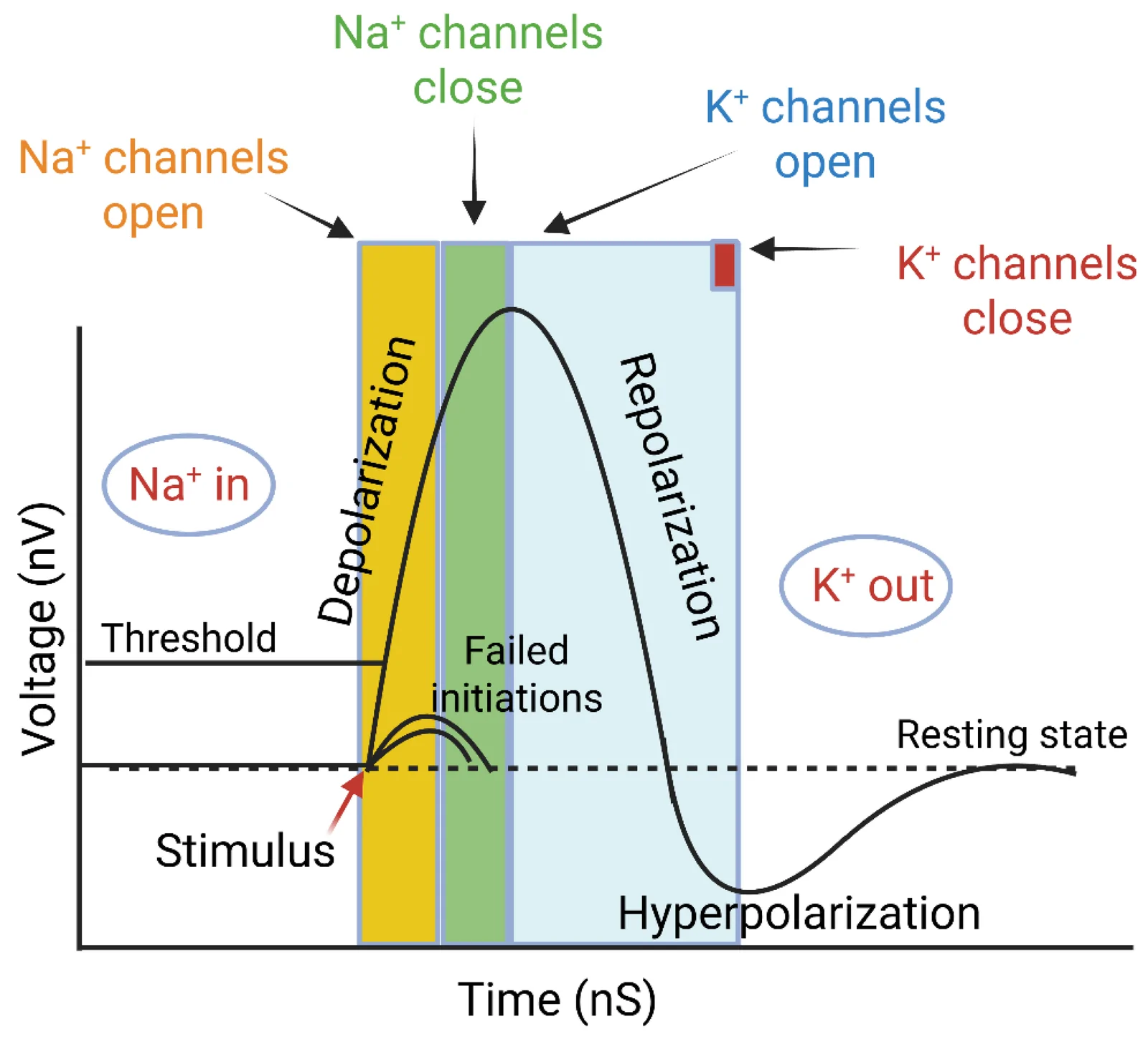
The hypothetical movement of Na^+^ and K^+^ through cell membranes. In this representation, cell membranes are depolarized first by Na^+^ (shown by the upward slope of the electrical wave), followed by the opening of slower-acting K^+^ channels and the diffusion of K^+^ out of the cell (lower membrane voltage, shown by the downward slope of the electrical wave). K^+^ channels close slowly, which means K^+^ continues to exit the cell after the membrane returns to its resting potential (zero potential). The figure was prepared using BioRender.com (accessed 2 June 2026).

The wave-like and tunneling behavior of quanta drives critical survival mechanisms, allowing microorganisms to harvest energy and communicate with remarkable efficiency. The binding of AIs to cellular receptors during QS relies on precise chemical shapes and electronic charges. In some cases, bacteria use multiple QS signals. The fundamental nature of these chemical bonds and hydrogen interactions is explained by quantum mechanics [27]. The rate at which cells produce (“fire”) successive waves of electrical signals depends on the kinetics of ion channels. The rapid sequence of voltage (potential) changes across the cell membrane is determined by the difference in ion concentrations across the membrane and the membrane’s permeability [28]. Because of the tunneling behavior of ions, they may, hypothetically, pass through a potential energy barrier even when they lack the classical kinetic energy to overcome physical obstructions [29]. This implies that ions in a quantum tunneling state could, theoretically, pass through bacterial membranes and cell walls in the absence of dedicated ion channels. This, however, does not imply that ion channels are not used. The hypothesis needs to be confirmed with biological experiments. Studies on neuronal cells have shown that if the cell membrane is in an excitable state, voltage-gated K^+^ channels (Kv channels) shape the electrical wave to form an action potential [28]. Bacterial cell membranes may behave similarly. However, this needs to be confirmed. Cell membranes are normally depolarized first by Na^+^ (observed as an upward slope of the electrical wave), followed by the opening of slower-acting K^+^ channels (Figure 6). Diffusion of K^+^ out of the cell lowers the membrane voltage, which explains the downward slope of the electrical wave seen in Figure 6. Potassium-ion channels close slowly [28], so K^+^ continues to exit the cell after the membrane returns to its resting (zero) potential. This implies that fluctuations in ion concentrations across cell membranes cause unpredictable variations in the positions, momenta, and energies of electrons, atoms, and molecules on both sides of the membrane.

Rapid variations in extracellular K^+^ gradients alter the motility of *Bacillus subtilis* cells and change their membrane potential [30,31,32]. The same behavior may apply to Cl^-^, Ca^2+^, and Na^+^ ionotropic glutamate receptors, enzymes, and larger molecules [26]. Fluctuations in ion concentrations and energy levels, a characteristic explained by quantum mechanics, introduce inherent randomness into biological processes such as DNA repair, enzyme reactions, and cellular signaling [28]. Larger molecules (e.g., protoporphyrins) and receptor-ligand interactions also exhibit wave–particle duality, quantum tunneling, coherence, and entanglement [33]. Subatomic particles and molecules that are in multiple energy states or conformations, thus in a “superposition” [34], allow rapid energy flow and more efficient molecular transfer than traditional chemical signaling. Theoretically, the coupling of particles across a distance, described as quantum entanglement [35], allows the transfer of energy between porphyrins and chromophore complexes [36]. This may give rise to complex biological structures, e.g., microtubules, transcription factors, and immune cells [37]. Theoretically, upon separation of the entangled particles, each particle returns to the same quantum mechanical state [37].

Hypothetically, this means biochemical reactions will proceed exponentially faster than classical thermodynamics would typically allow. This is, of course, a hypothetical concept, but it is still worth considering. The question is thus the following: Could this explain rapid communication among bacteria, especially over long distances? We argue that non-contact communication among bacteria rests on two pillars: QS and quantum mechanics/tunneling/entanglement. The wave-like behavior of subatomic particles and molecules leads to nanovibrations and the generation of electromagnetic fields. Could this explain how the direction of flagellar rotation is determined?

## 5. DNA Is Held Together by Quantum Harmonic Oscillations

**Figure 7:**
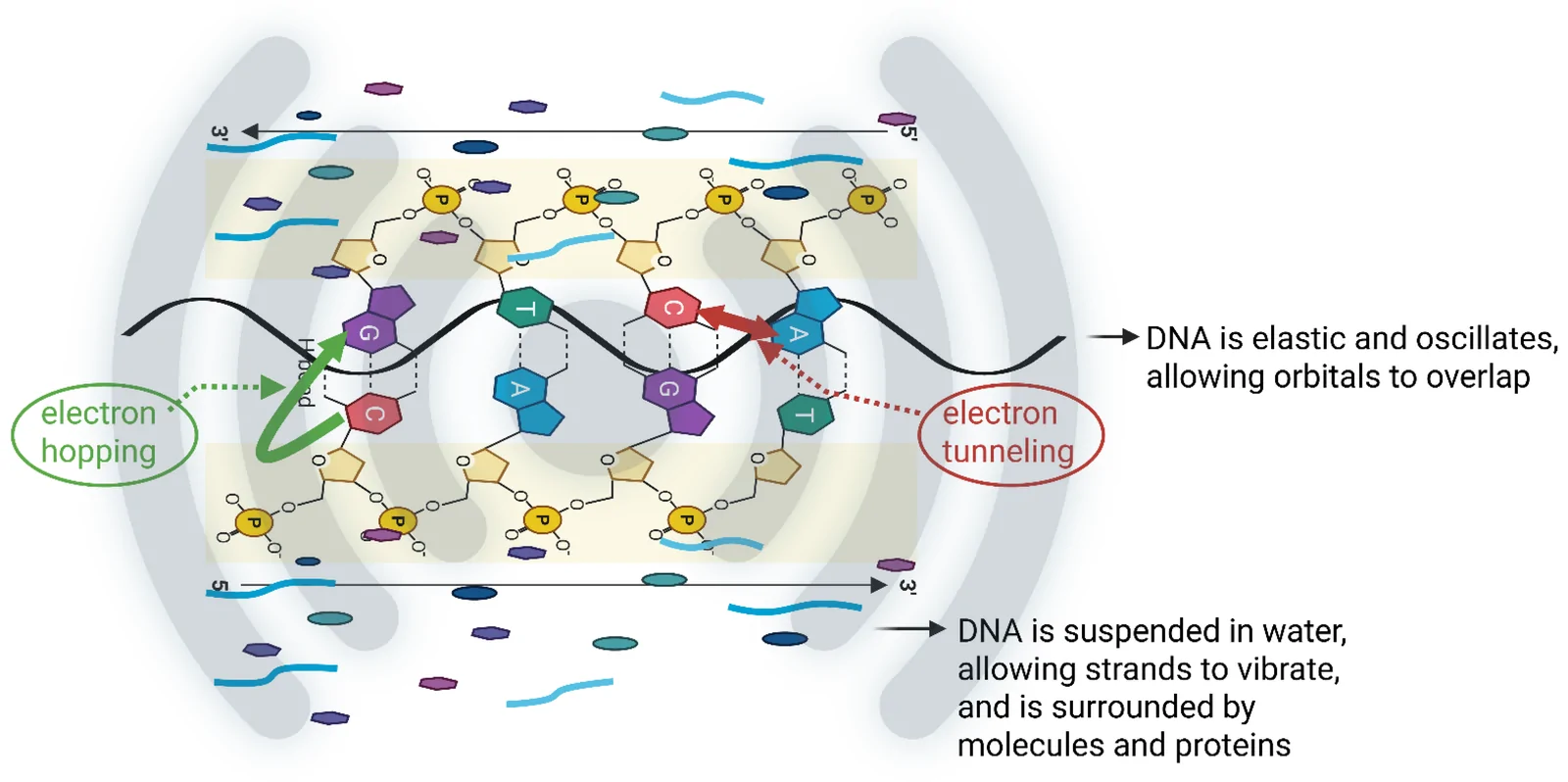
A representation showing the possible movement of excited electrons in DNA. DNA is elastic (flexible), suspended in the cytoplasm, and surrounded by molecules and proteins. The chiral structure of the DNA helix allows electrons with an opposite spin to be pushed in different directions along the chain. The overlap of close-by orbitals allows electrons to move from one orbital to another through quantum tunneling (red arrow). In orbitals not overlapping, electrons move by “hopping” (green arrow). Excited electrons are usually dragged towards guanine. Quantum tunneling may cause DNA to vibrate (depicted as expanding grey half-circles). The figure was prepared using BioRender.com (accessed 2 June 2026).

Hydrogen bonds link the base pairs across two DNA strands. Rieper et al. [38] described the van der Waals forces between stacked bases as dipole–dipole interactions (Figure 7) arising from overlapping “electron clouds.” This places molecules in a state of superposition. Proteins’ ability to identify consensus DNA sequences depends on the transfer of quantum information between nucleotide bases. Based on the model proposed by D’Acunto [39], the long-range nature of quanta allows nucleotide dimers to be recognized nanometers apart (the exact distance is unknown). This would allow repair enzymes such as DNA glycosylases to reach lesions in DNA strands much more quickly and efficiently. Excited electrons move by “hopping” (Figure 7) and enable enzymes to “slide” along DNA strands much faster via nonspecific binding while scanning the “normal” (undamaged) segments, bind to damaged sites, and rapidly repair the genome [40]. The DNA glycosylase MutY in *E. coli* recognizes rare 8-oxoguanine–adenine (oxoG:A) mismatches [41,42] and removes adenine from DNA. The [4Fe-4S] cluster in MutY, which grants the protein mixed-valent properties, undergoes a 2+ to 3+ oxidation when it couples to DNA, especially near oxoG [43]. The transfer of electrons stabilizes MutY’s binding to DNA, thereby enabling more efficient damage repair. To achieve this, the rate of electron transfer from the donor to the acceptor must exceed the normal one-dimensional sliding rate of MutY along DNA. This is only achievable if the electron transfer is quantum mechanically coherent. Lin et al. [44] reported that MutY is more prone to bind to oxoG than guanine due to the lower oxidation energy of oxoG. The authors also showed that with the [4Fe-4S] cluster in the 3+ state, MutY should bind DNA more tightly, stabilize specific binding, and allow the protein to remain in a recognition mode until it detaches from the DNA. These conclusions are based on multiscale calculations, including quantum mechanical (QM) calculations, molecular dynamics (MD), and the electron transfer pathway method. The hypothesis that long-range quantum communication networks within colonies trigger or upregulate DNA repair pathways, alter metabolic pathways, and regulate bacterial communication must be confirmed with biological experiments. Currently, definitive proof that macroscopic quantum networks (long-range electromagnetic fields and subatomic wave-like signals) drive these biological pathways and bacterial communication remains unestablished.

## 6. Can Neuroscience Teach Us Something About Bacterial Communication?

According to the theoretical orchestrated objective reduction (Orch OR) model proposed by physicist Roger Penrose and anesthesiologist Stuart Hameroff [45], human consciousness originates from quantum computations within microtubules. The authors argued that microtubule proteins act as quantum bits (qubits) when in superposition (in multiple conformational states simultaneously). Theoretically, the superposed microtubules become entangled and collapse when they reach a specific threshold. The collapsed state, referred to as the definite state, is when consciousness, or the “moment of awareness” and “choice,” forms. It is hypothesized that Orch-OR may be controlled or regulated by electrical synapses (gaps or junctions). This may, hypothetically, apply to bacteria. Cyanobacteria and *Shewanella oneidensis* use protein filaments (bacterial nanowires) to transfer electrons to external metals [46]. Ion channels in bacteria are structurally similar to those in the brain [47]. Sodium channels [48], chloride channels [49], calcium channels [50], and ionotropic glutamate receptors [51] used by bacteria are all voltage-gated. In the brain, disturbances in voltage-gated channels may result in epilepsy [52], pain disorders [53], and cardiac arrhythmias [54] (with the emphasis on may).

Qaswal et al. [55] have shown mathematically that quantum mechanics is a promising tool for addressing excitability-related diseases. The theoretical model described how ions permeate closed channels, explained the effect of hyperexcitability on potassium channels and the migration of Na^+^ through mutated channels, and shed light on the development of new agents to treat chronic and challenging diseases. The authors also speculated how certain ions can generate quantum membrane conductance and alter the resting membrane potential. Proton tunneling across hydrogen bonds between nucleotides in a DNA strand may alter base pairing, leading to spontaneous mutations [56]. This may very well also occur in bacteria. However, these hypotheses must be confirmed by biological experiments.

Bacteria are sentient. The concept is, however, highly debated and considered by some as speculative and a philosophical perspective. They know exactly what is going on in their environment and adapt accordingly to survive. Based on the hypothetical “two-pillar” model, bacteria may use QMTE in addition to QS to communicate with each other and with human cells. Traditional models rely strictly on the physical diffusion of molecules, whereas this hypothesis introduces instantaneous electrical and subatomic signals. Similar to excited (charged, fired) neurons, bacterial communities (colonies and biofilms) may communicate using bursts of electricity. The sudden change in electrical charge might place bacterial communities under stress, followed by the release of AIs once a specific cell density (quorum) is reached. It is hypothesized that when cellular signals untangle and cells return to a relaxed state, cellular structures, organelles, and DNA reorganize their internal energy into a single collective quantum state, thereby producing a definite conscious state accompanied by specific metabolic choices [57].

In response to the question posed by Hameroff and Penrose, we do not believe that bacterial cells in a colony or biofilm need to be synchronized or physically contact one another to alter their metabolic processes. This opinion is based on theoretical models and hypotheses and must be confirmed experimentally. Could metabolic co-dependence between peripheral and interior cells in a biofilm (perhaps on a smaller scale in a colony) generate quantum oscillations within the cell community? Synchronized collective oscillations may alter the potential of cell membranes and ion channels, thereby activating electrical communication [26,57]. Antiphase oscillations (time-sharing) might allow cells in each biofilm to take turns accessing the full quantity of supplied nutrients during their growth phase. Electrical impulses oscillating within a biofilm may attract distant cells and enable cross-species communication. For example, *Pseudomonas aeruginosa* cells are drawn to electrical signals emitted by *Bacillus subtilis* cells in a biofilm [30].

## 7. Conclusions

Quorum sensing (QS) is widely regarded as the most common method of communication among bacterial cells. However, electrical signaling may play a vital role in bacterial communication and gives rise to quantum mechanical phenomena such as tunneling and entanglement, albeit mostly based on hypotheses and speculations. Density-dependent subatomic particles (ions and electrons) that fall below a critical concentration threshold cluster into a single spatial region that behaves like a macroscopic condensate, known as the Fröhlich condensation, proposed by Herman Fröhlich. The Fröhlich model describes collective dipolar or phonon behavior in biological macromolecules at ambient temperatures, suggesting that cell membranes and proteins can, theoretically, sustain coherent vibrational states despite thermal noise. It is hypothesized that changes in electrical signals may cause subatomic particles, molecules, and cells to oscillate and propagate signals between distant biofilms. Quantum mechanics provides theoretical explanations for long-distance synchronization of processes such as biofilm formation and ion transport across cell membranes. These hypotheses must be confirmed by validated experimental studies. Quantum tunneling may place cells in a superposition state and may allow biofilms to form under limited nutrient supply. Several questions remain unanswered: Is quantum tunneling a common yet unobserved or underexplored process in the bacterial world? What environmental conditions trigger quantum tunneling, and to what extent? To what extent does quantum tunneling affect metabolic reactions in bacterial cells, i.e., are all genes affected by quantum tunneling? Do bacteria use quantum mechanics to survive harsh conditions, such as in the human gastrointestinal tract? Are there more molecules that behave according to the rules of quantum mechanics that we do not know about, and if so, how do they affect bacterial growth, cell division, and bacterial intelligence, cognition, consciousness, and decision-making? To what extent does quantum mechanics affect QS? Would it be possible to use quantum mechanics to alter QS? Further research on the proposed “two-pillar” system is required. Most scientists oppose claims that quantum tunneling influences bacterial communication, largely because of a lack of empirical evidence and validated biochemical/physiological experiments. However, the hypotheses provide a basis for future research, and the concepts should not be ignored. Future studies should use isotopes to track electron flow and measure changes in reaction rates. Nonlinear equations must be used to account for thermal noise in wet, warm environments. We need to demonstrate how specific enzyme structures or lipid arrangements can temporarily shield subatomic interactions from surrounding molecular chaos. Quantum states must be measured directly rather than inferred from macroscopic behavioral anomalies.

## Data Availability

No new data were created or analyzed in this study. Data sharing is not applicable to this article.
